# From Fighting Critters to Saving Lives: Polyphenols in Plant Defense and Human Health

**DOI:** 10.3390/ijms22168995

**Published:** 2021-08-20

**Authors:** Amber Stiller, Kendall Garrison, Karina Gurdyumov, Jacob Kenner, Farida Yasmin, Ping Yates, Bao-Hua Song

**Affiliations:** Department of Biological Sciences, University of North Carolina at Charlotte, Charlotte, NC 28223, USA; astille4@uncc.edu (A.S.); kgarri16@uncc.edu (K.G.); kgurdyum@uncc.edu (K.G.); jkeener6@uncc.edu (J.K.); fyasmin@uncc.edu (F.Y.); pyates4@uncc.edu (P.Y.)

**Keywords:** phytochemicals, chemical defense, crop improvement, disease prevention, immune system, anti-virals

## Abstract

Polyphenols, such as flavonoids and phenolic acids, are a group of specialized metabolites in plants that largely aid in plant defense by deterring biotic stressors and alleviating abiotic stress. Polyphenols offer a wide range of medical applications, acting as preventative and active treatments for diseases such as cancers and diabetes. Recently, researchers have proposed that polyphenols may contribute to certain applications aimed at tackling challenges related to the COVID-19 pandemic. Understanding the beneficial impacts of phytochemicals, such as polyphenols, could potentially help prepare society for future pandemics. Thus far, most reviews have focused on polyphenols in cancer prevention and treatment. This review aims to provide a comprehensive discussion on the critical roles that polyphenols play in both plant chemical defense and human health based on the most recent studies while highlighting prospective avenues for future research, as well as the implications for phytochemical-based applications in both agricultural and medical fields.

## 1. Introduction

The development of modern civilization and industrial practices has mediated severe and rapid climate changes resulting in global warming. Given their immobile nature, plants are incapable of migrating away from harsh environmental conditions and, therefore, must employ alternative strategies to deal with these challenges. Climate change impacts are variable and may include intensified or prolonged droughts, atypical fluctuations in seasonal temperature, and enhanced activity of herbivores. Enhanced plant stress contributes to concerns regarding global food security, which consequently is mainly supported by staple crops that supply humans with nutritious foods needed to sustain human health. In addition, climate change may alter the occurrence of infectious diseases by increasing the range, survival, and abundance of vectors that infect humans [1]. This has become a largely relevant concern with the recent emergence of the SARS-CoV-2 pathogen and resulting pandemic. Although plants encounter various environmental challenges, they possess a suite of defense mechanisms, such as the production of specialized metabolites, to protect themselves and ensure their survival. In turn, these specialized metabolites contribute to plant health and, potentially, extend to benefiting human health.

Plants can produce a diverse array of chemicals, broadly referred to as phytochemicals, that aid in key processes such as reproduction, growth, and defense [2]. These phytochemical compounds are divided into two major groups: primary metabolites and secondary metabolites. Primary metabolites are compounds utilized in plant growth, such as carbohydrates and amino acids. In contrast, secondary metabolites are produced, either constitutively or inductively, to protect plants from environmental stresses. Interestingly, many of the secondary compounds, such as isoflavonoids, can be utilized in combating various health-related issues in humans.

Phytochemicals are further grouped into several categories, of which, polyphenols (PPs) are the most prominent. There are over 8000 polyphenolic compounds, all of which are derived from a common precursor, phenylalanine, which is produced via the shikimic acid pathway and has been chemically modified for specific functions [3]. The major groups of polyphenols include phenolic acids, flavonoids and isoflavonoids, lignans, and stilbenes. Polyphenols, a crucial component in plant chemical defense, have largely mediated the coevolution of plants and their symbionts. Plants regularly encounter environmental stressors that trigger the transcription of enzymes used in PP production. Polyphenols aid in plant defense by accumulating in various tissues where they may neutralize free radicals produced in response to environmental stressors, or by increasing plant toxicity and unpalatability to herbivores and other organisms [4,5,6,7,8,9,10]. Following a stressful encounter and subsequent defense maneuvers, plants can re-allocate their resources toward growth and yields. With increased concentrations of PPs in plant tissues, plant fitness and nutritional values may be enhanced, which are critical in the improvement of plant and human health. When consumed, PPs utilized in plant defense may also be used by humans to reduce damage caused by oxidative stress and infectious diseases. For instance, the bitterness of a fruit or vegetable can deter herbivores from consuming the plant, but, conversely, may offer benefit for human health, as well as desirable tastes for human consumption with examples including the bitter flavor in coffee beans and glucosinolates in crucifer species [3]. Considering the growing concerns regarding human health, there is a need to enhance dietary nutrition and immune defenses via phytocompounds such as secondary metabolites. As such, these phytocompounds may provide a diverse array of benefits through regular consumption of plant-based foods, making them reasonably affordable and accessible.

Although significant progress has been made in linking plant health and human health benefits, there are still many unknowns regarding this area of research. Regular consumption of fresh fruits and vegetables has been shown to significantly reduce the risk of developing chronic diseases, such as cancer and diabetes [11]. Therefore, plants may contribute to long-term human health by reducing the incidents of illnesses and onset of diseases, thereby, reducing the need for medicinal treatments. Furthermore, expanding our knowledge of plant health is crucial in determining how plants and their phytochemicals can be utilized to benefit human health. Enhanced vulnerability to diseases, herbivore pests, and competition, may drastically reduce agricultural crop yields, resulting in diminished food availability and accessibility worldwide. In light of these concerns, understanding the role of PPs and other phytochemicals in plant health may facilitate agricultural practices and selective-breeding strategies to promote crop yields and traits.

**Figure 1:**
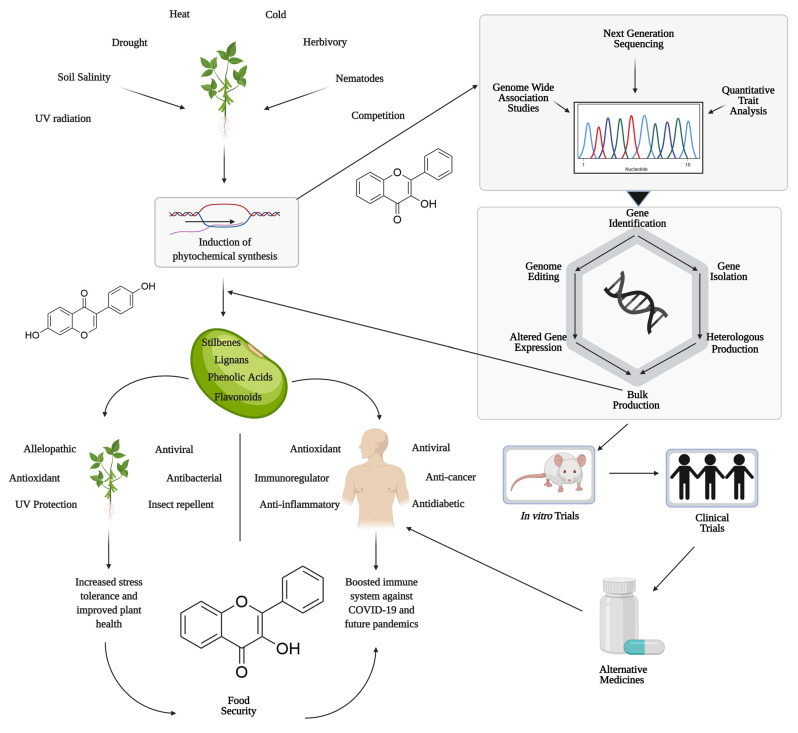
A schematic representation of polyphenols in both plant chemical defense and human health and how modern technologies can be utilized to enhance production of these compounds (Created with BioRender.com).

This review aims to provide a comprehensive overview of various PPs and their mechanistic actions in plant defense and potential functionality in human health. While previous review articles mainly focused on the biological activity of PPs in humans, this review will discuss the most recent progress in both aspects, as well as challenges and perspectives in this field. Figure 1 illustrates the roles of polyphenols in both plant chemical defense and human health and various technologies that can be utilized to enhance the production of these compounds.

To collate information regarding the use of polyphenols in plant defense and human health, online databases were utilized to search for peer-reviewed articles. These databases included PubMed and Web of Science. Searches were confined to research published between 2015 and 2021, except for some classical articles cited in the introduction section.

## 2. Roles of Polyphenols in Plant Health

The production of PPs greatly improves plant fitness in the presence of abiotic and biotic stressors. Abiotic stressors, such as drought, UV radiation, temperature fluctuations, soil salinity, and heavy metal exposure, can reduce plant growth and yields by inducing increased production of reactive oxygen species (ROS) that destabilize and damage plant organelles and macromolecules [4,5,6,7]. PPs act as free radical scavengers that neutralize ROS, which helps maintain environmental homeostasis and plant resiliency [4,5,6,7,8,9,10]. Alternatively, allelopathic interactions associated with biotic stressors, such as herbivorous insects, parasitic nematodes, and plant competitors, may also induce ROS production, resulting in damage to plant tissues [12,13,14]. Elevated activity of PPs may increase plant resistance and thereby provide a competitive advantage over plants harboring fewer PPs. Collectively, biotic and abiotic stressors sequentially and simultaneously contribute to the generation of ROS, which may be neutralized by many of the same PPs that help alleviate multiple stressful conditions. For example, tannins help alleviate drought and salinity-induced stress but also participate in herbivore resistance, all of which serve as competitive advantages for plants [12,15,16,17,18,19,20,21]. Plants subjected to a combination of abiotic and biotic environmental stressors may develop greater overall resilience to harsh environmental conditions due to the presence of various PPs as opposed to plants subjected to a single stressor at a time [22]. While this review discusses various aspects separately, many concepts will reoccur across multiple sections due to the interrelated nature of various topics. The following section discusses the roles of PPs in plant abiotic stress tolerance and biotic stress resistance as it applies to enhanced plant health.

### 2.1. Polyphenols in Abiotic Stress Tolerance

#### 2.1.1. Drought

Although 71% of the Earth’s surface is covered with water, drought is still a significant factor limiting plant productivity, which will likely play a more prominent role as climate change effects become increasingly severe. Drought-induced stress stimulates increased production of abscisic acid (ABA) in plant tissues, a phytohormone that induces stomatal closure by depolarizing guard cells and decreasing turgor pressure in the surrounding tissues [16,23,24,25,26]. Furthermore, some studies suggest that ABA only keeps the stomata closed, which aids in water conservation and prevents further vascular system damage [27]. However, this closure can result in the deprivation of carbon dioxide, a key molecule required for photosynthesis, which is utilized in the Calvin cycle, where electron acceptors are produced [16,23]. In the event of low availability of electron acceptors, free radicals accumulate in plant tissues and may induce tissue damage. To neutralize these free radicals, the phenylalanine metabolic pathway is activated to enhance PP production [15,16,23,28,29]. The most common PPs upregulated during drought include anthocyanin and other flavonoids (tannins, stilbenes) [15,16]. Increased PP production in response to drought has been observed in various plants, including grape, rice, peanut, fern, wheat, and various shrubs [15,16,23,24,25,29,30].

Drought-tolerant plants produce more PPs in comparison to drought-intolerant plants [5]. Additionally, drought-tolerant plants synthesize PPs more quickly than intolerant plants, which may help buffer the effects of ROS while partitioning more energy for photosynthesis and growth rather than stress relief [24,30,31]. This observation could be due to higher sensitivity to ROS and other signaling pathways that trigger water conservation mechanisms. Given the distinct composition and concentration of PPs produced by plant cultivars, farmers may consider implementing polycultures to produce more resilient crops while maintaining a diverse group of PPs.

#### 2.1.2. Temperature Variation: Heat and Cold Stresses

Temperature-induced stress due to extreme changes in temperature can detrimentally impact agricultural productivity for many types of plants worldwide. Both heat and cold stresses contribute to the production of ROS, most of which are generated from the chloroplast where photosynthesis may be altered via the electron transport chain [32]. Similar to drought-induced stress, elevated temperatures can lead to the closure of stomata, and contribute to photoinhibition and increased membrane permeability, which may reduce water and solute retention in plant cells [33]. The ROS produced from these processes can be scavenged by a variety of PPs upregulated in response to elevated temperatures, including anthocyanin, flavonols and other flavonoids, tannins, and phenolic acids [5,22,34]. Additionally, heat stress may upregulate the synthesis of p-coumaric, ferulic, and caffeic acids that seal plant cells to reduce membrane permeability and leakage [34]. Similar to elevated temperatures, lower temperatures can result in ROS production and adverse cellular effects. These similarities suggest that cold and heat tolerance mechanisms may be fairly similar [5,33]. ROS produced in response to low temperatures are scavenged by various phenolic acids, flavonoids, and tannins [5,35,36]. During cold conditions, enhanced PP content and longevity may be attributed to glycine betaine, a molecule that increases enzymatic activity of phenylalanine ammonia-lyase (PAL) for long periods of time, resulting in increased long-term cold tolerance in plants [33,36].

#### 2.1.3. UV Radiation

Due to increased greenhouse gas emissions and reduced ozone levels, exposure to UV radiation has become an increasingly relevant issue for plant and human health. UV radiation promotes DNA damage via ROS production, following which, PP defense mechanisms are activated [37], resulting in enhanced PP synthesis and increased PP levels being translocated to UV-damaged areas [38,39]. Two major functional categories of PPs are synthesized in response to UV radiation: absorbing and shielding [5,15,38,40,41,42]. Absorbing PPs, mostly flavonoids, accumulate in the mesophyll of leaves, where they scavenge ROS produced by absorbed UV light [38,42]. On the other hand, UV shielding flavonoids accumulate in the epidermis of leaves, where they act as blocking agents or by reflecting UV light [41]. Typically, shielding PPs are most abundant during midday conditions, whereas absorbing flavonoids are most abundant during morning and evening [38,42]. After being synthesized, PPs are translocated from other plant tissues to damaged areas, which could have implications for human health benefits depending on which plant tissues are consumed and the temporal localization of PPs. Therefore, altering the harvest time (time of day or season) to ensure that PPs are localized in specific plant tissues of interest may be beneficial to commercial farmers and could be implemented in future agricultural practices. While UV light does generate ROS that are harmful to cells, exposure to UV light has also been shown to increase PP levels in the fruits of plants [43,44]. For example, in apples exposed to UV radiation, the apple epicarp exhibits much higher concentrations of anthocyanins and flavonoids when compared to normal conditions [43]. Furthermore, UV-radiated apples also develop heavier fruit in comparison to apples grown in shaded conditions [43]. Heavier fruits may be partly attributed to higher concentrations of specialized metabolites induced via UV light stress [45]. Moreover, reduced UV stress by one PP may simultaneously promote plant tolerance of other environmental stressors, such as herbivory and salt stress [46]. Some notable PPs that exhibit increased concentrations in response to UV radiation include caffeic acid, flavonoids, and ferulic acid [5,40,43,44].

#### 2.1.4. Soil Salinity

Salinity stress impacts various plant physiological processes, including their involvement in regulating turgor pressure, which supports overall plant growth and health [17]. However, increased salt content may induce photosynthetic stress by generating ROS and interfering with key processes [17,18]. For example, exposure to high-salinity conditions in plants alters electron transport chain activity and light harvesting complexes, resulting in ROS production [18]. To protect their photosynthetic machinery from ROS-induced damage, plants increase PP concentrations in their leaves and stems [17,18,29,45]. In addition to producing a distinct array of PPs varying in concentration, different plant species demonstrate varying tolerance levels to environmental stressors. For instance, distinctive salt tolerances are exhibited by two halophytes, common reed (*Phragmites karka*) and sea rocket (*Cakile maritime*) when exposed to high-salinity conditions, resulting in varying PPs concentrations with PPs levels being lower in common reed than in sea rocket [18]. Furthermore, it is hypothesized that PPs synthesized by common reed possess more potent antioxidant properties than those synthesized by sea rocket [18]. Additionally, moderate-salinity conditions may enhance overall PP content and plant biomass [17,18,47] by enhancing membrane protection via the activity of flavonoids and other antioxidants such as proline, anthocyanins, and carotenoids [17]. At a certain point, elevated salt stress no longer promotes enhanced PP concentrations but results in reduced PP content and declining plant health [17,47]. Soil salinity is a major problem in agriculture, particularly regarding high evaporation rates. Agricultural management practices could employ salt-induced stress to promote threshold stress levels associated with specific cultivars to enhance plant biomass and polyphenolic content, which contribute to overall plant health and human health benefits. These management practices may be implemented in areas where soil salinity is negatively impacting crop yields. For example, the implementation of cover crops, aimed at replenishing and rebuilding soil health, and crop rotations could help reduce the need for synthetic fertilizer application and, therefore, help manage soil salinity and promote plant health.

#### 2.1.5. Heavy Metal Pollution

Heavy metal pollution caused by increased use of phosphate fertilizers and expanded industrialization is one of the growing concerns within the agricultural industry [48], which is strongly linked to anthropogenic factors. Although heavy metals may be present in the soil, significant uptake by plants can only be accomplished when the soil becomes acidic [49,50], which can occur through increased anthropogenic inputs (nitric oxides and sulfur dioxides) that precipitate acid levels in soils [49]. In addition to enabling heavy metal absorption in plants, acid precipitation promotes nutrient leaching in soils, leading to nutrient deficiencies in plants [31,49,51]. Furthermore, heavy metal uptake may reduce plant metabolic rates by interfering with key biological processes such as photosynthesis, respiration, mitosis, and membrane permeability to macro and micronutrients [31,49,52]. The onset of heavy metal stress can induce PPs as plant defense mechanisms, known as phytochelatins, which include ascorbic acid, flavonoids, and many other phytochemicals [31,48]. These PPs have specialized structures that help mediate ROS reduction, binding of metal ions, and production of monooxygenase [31]. Monooxygenase, an enzyme involved in the microsomal system and cytochrome P450 activity, plays a major role in detoxifying xenobiotics such as heavy metals [31,53]. Collectively, the implementation of various agricultural practices, such as cover crops, crop rotations, and, when appropriate, lime application, could potentially promote optimal environmental conditions that stimulate PPs levels and support plant growth and health. Specifically, cover crops and crop rotations may reduce the need for fertilizers, while lime application may reduce heavy metal uptake by increasing soil pH when needed.

### 2.2. Polyphenols and Biotic Stress Resistance

#### 2.2.1. Herbivory

Plants are continuously exposed to herbivorous threats in their environment. Herbivores inflict damage to plant tissues, which initiates the production of ROS and phytohormones that signal plant defense mechanisms [12,13]. Similar to their response to abiotic stressors, plants produce PPs in response to biological stressors such as herbivory. Various herbivore activities can trigger plant defense, including inflicted tissue wounds, stylet injection, oviposition, chemical injection, and vibrations [13,54,55]. For example, the production of the phytohormone jasmonic acid (JA) may be stimulated at the site of tissue damage and initiate a signal cascade that results in the production of PPs [54,56]. PPs can be detrimental to herbivore fitness by interrupting egg development and reducing fecundity of parent herbivores that ingest them [57]. Furthermore, PPs, such as phenolic acid and flavonoids, can reduce larval weight gain and result in a lengthened developmental period [12,57]. The reduced weight gain may be attributed to the anti-feedant properties of PPs that can result in malnourished insect larvae and delayed insect growth [57]. Plants may also employ indirect defense mechanisms by releasing volatile PPs, such as methyl salicylate, into the atmosphere to attract predators of the feeding insect to the host plant [19]. Volatile compounds can be released in response to plant wounding or herbivore-induced vibrations [13,19,58]. PPs that aid in herbivore resistance include phenolic acids, chlorogenic acid, flavanone glycoside, quercetin, tannins, 3-D flavonoids, and many other flavonoids [12,19,20,21,59]. While the individual actions of PPs, such as chlorogenic acid, may have limited effects on herbivore activity, the synergistic actions of these PPs may elicit greater effects on herbivores, thereby bolstering plant defense [60]. In contrast, certain phytochemicals, such as chlorogenic acid and pyrrolizidine alkaloid free bases, may exert antagonistic effects that are counterproductive to plant defense when utilized in combination and, therefore work better individually [60]. Additionally, plant resistance to one herbivore may increase its resistance to another herbivore [46,61]. For example, infestations in potato tubers by the Guatemalan tuber moth results in increased chlorogenic acid concentrations in foliar tissue which aids plant defense against aboveground herbivory but not against tuber moth [61]. It is hypothesized that this observation is due to the adaptation of tuber moths to plant defense mechanisms [61]. In addition, aphid infestations in tobacco can result in salicylic acid production, which in turn, may aid in pathogen defense but not directly contribute to aphid resistance [11].

In addition to affecting insect physiology, plant defense mechanisms may alter insect behavior. Although insect interactions with plant tissues generally cause physical damage, plant chemical defenses may, in turn, alter insects’ interactive behaviors in ways that benefit plants themselves. Although plant chemical defenses continue to evolve to become more effective against herbivore activity, insects continuously coevolve alongside plants by developing their own defense mechanisms and selective resistance against phytochemicals. For instance, insects have been observed to insert their stylets at shallower depths in plant tissues containing high amounts of PPs [20]. Alternatively, insects may intentionally avoid interacting with plants that have accumulated PPs [62]. This suggests that phytochemical defense against herbivores may assume both constitutive and induced defense strategies. In addition to modifying their behavior, insects have evolved mechanisms to sense the chemical compounds in plants to determine which plants are suitable for egg deposition and offspring survival [59,62]. This suggests that herbivore-induced PP production not only is involved in plant defense, but also influences co-evolutionary trends that determine organism behaviors and/or anatomical changes in response to phytochemicals. This observation in particular has implications for agricultural practices as some synthetic insecticidal compounds are structurally similar to that of naturally-occurring phytochemicals [63]. As insects coevolve with plants to become increasingly resistant to phytochemicals, similarly, insects simultaneously acquire resistance to synthetic insecticides commonly utilized in agricultural practices [63]. As a result of these chemical similarities, the utilization of synthetic chemicals may accelerate herbivore resistance to plant defense compounds with little to no change in plant phytochemical adaptations in response to this resistance, therefore, interfering with plant defense mechanisms while enabling higher levels of insect herbivory.

#### 2.2.2. Nematodes

In addition to continuously encountering abiotic and biotic stressors aboveground, plants may also encounter threats belowground, such as parasitic nematodes. The presence of soil nematodes can present several challenges for plants, including stress associated with damaged vascular tissues resulting in nutrient deficiencies and water loss [64]. Furthermore, nutrient deficiencies can hinder plants from synthesizing key molecules needed to generate PPs, making plants more susceptible to herbivory, disease, and parasitic nematodes [65]. As suggested by Gao et al., the application of phosphorus fertilizers may help improve crop resilience against nematodes by supplying elements needed for PPs synthesis [65]. However, further research should investigate the efficacy of alternative and more sustainable methods. It is well established that plants respond to nematodes, both directly and indirectly, via the production of PPs [65]. For example, plants may deter nematode infestations by producing flavonoids and anthocyanins to reduce the palatability of root tissues [64,66,67]. Furthermore, plants release phytochemicals that act as allelochemicals, such as salicylic acid and cinnamic acid, into the rhizosphere to hinder nematode and pathogen activity [65]. For example, the release of cinnamic acid significantly reduces nematode growth, while the release of salicylic acid helps shield the plant from harmful toxins secreted by pathogens [64,65,66,67]. Alternatively, phenolic acids may inhibit nematode activity by interfering with nematode signaling pathways leading to nematode mortality [65].

#### 2.2.3. Competition

Plants compete with neighboring plants for limited resources, including water, nutrients, space, and sunlight. In particular, the spread of invasive plant species such as knapweed, garlic mustard, and mimosa, is problematic for native plants [14,68] that may be outcompeted by invasive species, making native plants vulnerable to population declines or extinction [14,69]. Invasive species may produce novel phenolic allelochemicals, such as filifolinol, filifolinone, and gallic acids, those native plants have not yet developed any resistance to, leading to an ecosystem less habitable for native plants or desired crop species [14,70,71]. However, native plants, such as the black walnut (*Juglans nigra*), may respond to allelochemical threats from invasive plants by producing their allelochemicals, such as juglone, to create a microecosystem less hospitable for plant competitors [72]. Belowground, the presence of soil PPs, such as flavonoids, phenols, and tannins, can induce ROS production and decreased nutrient permeability in plants [68]. Furthermore, many allelopathic effects elicited by plants demonstrate target specificity. For instance, a particular type of flavone, produced by tropical bracken fern, has allelopathic effects on a species of sesame (*Sesamum inducum*) [73]. Due to the targeted nature of this mechanism, species-specific allelopathic compounds have gained recognition as potential candidates for natural herbicides in agricultural settings [71,72,73]. However, some studies demonstrate that continuous monoculture crops exhibiting strong allelopathy can result in autotoxicity for new seeds of the same species [74]. Moreover, implementing effective, integrative pest management practices could potentially alleviate this issue and further contribute to weed suppression, improved soil health, and decreased toxicity which could enhance overall crop yields.

### 2.3. Multi-Stress Response and Phytochemical Interaction

Given that plants are exposed to multiple stressors simultaneously, plants have adapted to prioritize which stressors are most imperative to address, resulting in trade-offs between plant health aspects [75]. This process is mediated by ‘cross-talk’ between signaling molecules, such as JA and SA, which act antagonistically or synergistically based on the threat to fine-tune the desired defense strategy [75]. For example, plants infected by a specific bacterium may be more susceptible to a certain fungal species due to signaling molecules produced by the bacteria that suppress the plant’s own signaling molecules induced by the fungus [75]. This supports recent research detailing the altered composition and concentration of PPs produced by plants under multiple stressors [22]. On the other hand, plants may also cope with various stressors simultaneously. For example, in rice, UV radiation is known to induce the production of phenolic acids, such as caffeic acid, to protect their tissues from damage, while additionally functioning as allelochemicals to suppress the activity of nearby weeds [74]. The complex, and ever-evolving interactions among plant defense mechanisms have provided many benefits to plants, particularly relating to the diversity and functionality of their phytochemicals which are crucial for plant defense and overall health. Understanding the interaction of phytochemicals in plant tissues under stress will help elucidate the benefits of these compounds and their potential antagonistic or synergistic interactions.

In summary, a variety of environmental stressors (biotic and abiotic) can activate plant defense mechanisms and alter PPs concentrations and composition in plant tissues. After being synthesized, PPs are translocated to specific plant tissues where they accumulate and help alleviate specific stress-induced effects. Notably, PPs facilitate plant defenses against abiotic-induced stress (ROS) and biotic-induced stress (herbivory, nematodes), all of which may significantly improve plant health, crop yields, and potential for human health benefits. Table 1 summarizes the recently studied examples of PPs in plant stress responses.

## 3. Polyphenols in Human Health

In addition to their multi-functional roles in plant health, PPs also play important roles in human health by offering various potential health benefits regarding disease prevention and treatment. The section below discusses the benefits of PPs in diverse areas of human health. Table 2 summarizes the role of various polyphenols in human health.

### 3.1. Antioxidants

Oxidative stress triggered by free radicals may lead to the development of chronic diseases [90,91,92]. In addition to destabilizing and damaging plant organelles and macromolecules, ROS are also involved in cell differentiation, progression, and apoptosis in humans [90,93]. Many of these effects may be attributed to ROS interactions with membrane lipids, nucleic acids, and proteins in human cells [90,91]. Elevated ROS levels may reduce immune system activities and contribute to the onset of various human diseases, such as inflammatory diseases, cancer, and diabetes [90,91]. Antioxidant properties, characteristic of most PPs, may help protect cells from oxidative damage caused by ROS [90,91,93,94], as well as induce other biological activities such as anti-inflammatory, anti-cancer, anti-diabetic, and anti-viral properties [77,91,93,94,95,96].

The most notable PPs known for their antioxidant activity are catechins, which are predominantly found in green tea and black tea but also in various fruits [97,98] (Table 2). In comparison to other known antioxidants, catechins exhibit the most potent antioxidant potential due to their ability to react with a broader range of ROS [97,99]. As powerful antioxidants, catechins may help neutralize ROS within cells and help protect red blood cells (RBC) from membrane damage [97,99,100]. Furthermore, the unique structural and chemical properties of catechins enable them to permeate RBC membranes and spread throughout the phospholipid tails, reducing ROS entry and potential cell damage [97]. Red blood cells are particularly vulnerable to free radical attacks given that they transport oxygen molecules and have highly permeable membranes [97]. As a result, catechins may offer the most protective benefits to RBC that encounter free radicals more frequently than other cell types [97].

While catechins reportedly act as antioxidants in RBC, other studies report that PPs also demonstrate pro-oxidative effects that counter the activity of other antioxidants. For example, a study by Tedesco et al. found that PPs shift from antioxidants to pro-oxidants in erythrocytes [101]. Additionally, studies suggest that the pro-oxidant activity of PPs may better equip RBC by activating antioxidant enzymes, giving cells an adaptive advantage to defend against enhanced ROS concentrations in the future, a mechanism described as para-hormesis in which naturally non-toxic compounds mimic damaging compounds to stimulate other beneficial effects [101,102]. Further investigation of PP activity in RBC should be conducted to determine the extent to which PPs act as antioxidants, as illustrated by Grzesik et al., and pro-oxidants, or whether different PPs act antagonistically in this cell type with some PPs acting as antioxidants while others act as pro-oxidants [97,101]. Interestingly, the same PPs may exhibit tissue state-specific functions in the human body [93,100,101]. For example, in healthy tissues, PPs play a role in scavenging free radicals, thus acting as antioxidizing agents [93,100]. In contrast, in cancerous tissues comprised of elevated copper ion concentrations, PP functionality may shift from antioxidants to pro-oxidants that block other antioxidant enzymes [93,100]. Additionally, this switch in the role of PPs in unhealthy cells may promote health benefits by accelerating apoptosis in cancerous cells [93]. Further research investigating the roles of PPs in different cell types and conditions is needed to determine whether PPs can be utilized to improve the state of multiple cell/tissue types under various conditions.

To further add to the complexity of PP chemical activities, PPs, such as flavonoids, can also interact with other phytochemicals, such as carotenoids or vitamins that coexist in plant tissues. These compounds possess distinctive chemical properties that may impact how they interact with biological membranes. For example, the hydrophilic and lipophilic properties of PPs and carotenoids, respectively, may alter their interactions with one another or with cell membranes, resulting in strengthened or weakened antioxidant functionality in plants and humans [103,104]. More specifically, PPs can interact with other phytochemicals via chemical bonding (conjugation) or by occupying spaces near other phytochemicals, such as the water-lipid interface in cells (liposome) [103,104]. Studies show that conjugates of silybins with retinoic acid have greater antioxidant activity on certain free radicals while simultaneously demonstrating lower scavenging activity against other types of free radicals when compared to non-conjugated parental molecules [103]. This observation may be attributed to enhanced electron donating activity induced by intramolecular proximity [104]. Furthermore, the antioxidant activity of PPs and carotenoids that occupy the same or neighboring cellular spaces, such as liposomes, may exert greater antioxidant potential due to enhanced electron donation from anionic PPs to cationic carotenoids [104].

### 3.2. Anti-Inflammatories

#### 3.2.1. Neurological Pathways

Flavonoids comprise the largest group of dietary PPs which are further grouped into subcategories, including anthocyanins, flavonols, and flavones [3,70,77]. Flavonoids possess strong anti-inflammatory properties that may target brain cells and neurological pathways [3,70,77,105,106]. In doing so, flavonoids reduce deficits in motor and cognitive behavior due to aging and neurodegenerative diseases, such as dementia [77,105,106]. Flavonoids may elicit these effects by reducing neuroinflammation and oxidative stress in the neuronal environment [70,77,106]. As reported by Shukitt-Hale et al., juvenile mice supplemented with flavonoid-rich blueberries exhibited increased striatal and hippocampal protein kinase Cα (PKC) due to reduced neuroinflammation [77]. PKC is crucial for the regulation of synaptic plasticity, the production of growth factors that stimulate neurogenesis, and the conversion of short-term to long-term memory [77,107]. In adult mice, a diet composed of various flavonoids and curcumin promotes increased levels of extracellular signal-regulated kinase (ERK), which, similarly to PKC, correlates with increased neurogenesis, the generation of neurons from neural stem cells [77,106,108]. Furthermore, ingestion of flavonoids shows improved short-term and long-term memory, suggesting stronger neuronal communication and continued neuron generation into adulthood [77,106]. In turn, strong neuronal communication and generation may help prevent the onset of neurodegenerative diseases [77,106].

#### 3.2.2. Chronic Inflammation

While curcumin possesses antioxidant and anti-inflammatory properties similar to flavonoids, curcumin may help target inflammation related to chronic diseases and conditions such as arthritis [76,109]. Tumor necrosis factor α (TNF-α) is a cell signaling molecule responsible for inflammation in chronic disease and is regulated by the transcription factor NF-κB, a protein that significantly alters gene expression while increasing inflammation [76,110]. Polyphenols such as curcumin and fisetin ultimately inhibit NF-κB activity, which is activated by pro-inflammatory stimuli, leading to the reduction of TNF-α expression and, therefore, inflammation [76,110]. It is thought that the reduced NF-κB activity via curcumin consumption involves pro-inflammatory cytokines, such as interleukins, and reduced activity of pro-inflammatory enzymes, such as myeloperoxidase [110,111]. Another example of beneficial effects of PPs includes their pro-inflammatory impact on skin cells exposed to UV radiation [112]. Following exposure to UV radiation, several cellular events occur that can threaten human health: activation of NF-κB activity and production of ROS [112]. As stated previously, NF-κB acts as a pro-inflammatory factor in humans that is regulated by the interaction of PPs with cytokines [76,112]. However, ROS also plays a role in cellular oxidative stress regulated via the Nrf2 transcription system in human cells [112]. Interestingly, study shows that ingestion of PPs in combination with carotenoids, another group of phytochemicals, may help resolve these two processes simultaneously [112]. Furthermore, the combined activity of PPs and carotenoids in the human body has shown promising results regarding NF-κB inhibition in individuals exposed to UV radiation [92,112]. The ROS scavenging capacity, regulated via the Nrf2 transcription system, is significantly greater when PPs and carotenoids work concurrently than individually [112]. This finding suggests that, similarly to plant defense, PP activity may be altered by interactions with other phytochemicals.

In addition to defending against UV-induced inflammation, daily curcumin supplementation may help alleviate joint pain and increase joint functionality affected by mild to moderate knee osteoarthritis [76]. Similarly, another study comparing curcumin supplementation to nonsteroidal anti-inflammatory drugs (NSAIDS) found that curcumin provides the same anti-inflammatory and pain-relieving benefits as NSAIDS when treating osteoarthritis. Furthermore, the group treated with NSAIDS reported gastrointestinal issues while the group supplemented with curcumin did not experience these negative side-effects [76], suggesting that curcumin could be utilized as a more appealing alternative treatment to NSAIDS (Figure 1).

### 3.3. Anti-Cancers

#### 3.3.1. Breast Cancer

Recent research has garnered interest in evaluating the anti-carcinogenic effects of lignans (Table 2). After ingestion, plant lignans are metabolized by the gut microflora and converted into mammalian lignans for efficient absorption, which may help reduce the risk of various cancers, including colon, prostate, and breast cancer [80,113]. Lignans impact cancer cells via two different mechanisms: interference with cell division pathways and exertion of phytoestrogen properties [80,81,113,114]. A specific lignan, kusunokinin, demonstrated the ability to bind to five different target proteins involved in breast cancer progression, as reported in a study by Rattanaburee et al. [114]. These specific proteins suppress breast cancer cells by inhibiting cell proliferation, cell signaling, and cell invasion of cancerous cells [114]. Additionally, lignans have been utilized as a treatment for breast cancer due to their phytoestrogen properties [80,113]. Phytoestrogens regulate estrogen production in the body, which may reduce the risk of estrogen-related cancers, including some types of breast cancer [80]. Additionally, as reported by McCann et al., lignan intake in post-menopausal women diagnosed with breast cancer significantly reduces mortality rates in cancer patients, as well as patients with other illnesses [81]. Furthermore, reduced risks for breast cancer may be observed in both pre- and post-menopausal women following lignin intake [80].

#### 3.3.2. Colorectal Cancer

Phenolic acids elicit anti-carcinogenic effects in the body similar to that of lignans. Both of these phytocompounds are digested by gut microbiota and converted into microbial metabolites [83,115]. Many phenolic acids elicit antioxidant and anti-inflammatory properties, both of which may contribute to lowered risks of cancer [83,116]. Specifically, phenolic acids may help regulate signal transduction pathways between cells, inhibit cancer cell growth and proliferation, and prevent cell damage from free radicals [83,115]. Most antioxidant and anti-inflammatory properties elicited by phenolic acids have the most potential to benefit the colon, where most phenolic compounds remain undigested [83]. Following this, the colon’s microbiota may break down PPs and enhance their bioavailability, potentially contributing to reduced risks of colon cancer [83], as observed in a study consisting of over 48,000 men and women [84].

### 3.4. Anti-Diabetics

Tannins are another type of PPs that exhibit various health benefits such as anti-inflammatory, anti-carcinogenic, anti-viral, and anti-diabetic properties [88] (Table 2). More recently, the anti-diabetic properties of tannins have been shown to relieve symptoms of high glucose levels found in both type-1 and type-2 diabetes [88]. These symptoms are classified as diabetic nephropathy, diabetic retinopathy, diabetic neuropathy, and diabetic cardiomyopathy [88]. Hyperglycemic conditions leading up to diabetic nephropathy and retinopathy may stimulate various pathways that increase ROS production, which may lead to blood vessel damage and leakage [88]. Furthermore, the antioxidant activity of tannins may help mitigate the formation of ROS by interfering with various pathways involved in its production [89]. Additionally, hyperglycemic conditions may damage peripheral nerves and spread to other parts of the nerve system via the spinal cord, leading to diabetic neuropathy and sensory loss [88]. In conjunction to acting as pain relievers, tannins also may help prevent ROS-induced damage in neurons and make repairs [88]. Diabetic cardiomyopathy has a high mortality rate among diabetic patients as it greatly increases the chances of developing heart disease [88]. Diabetic cardiomyopathy is associated with the accumulation of glucose in plasma and cardiac tissue [88]. However, tannins have been shown to help elevate insulin levels, leading to decreased glucose levels in plasma and cardiac tissue [88].

Resveratrol (Table 2), a PP grouped as a type of stilbene, aids in the prevention of type-2 diabetes by eliciting strong anti-diabetic properties [85,86]. Like tannins, resveratrol may help inhibit diabetic cardiomyopathy but does not affect insulin levels [87]. More specifically, resveratrol helps inhibit cardiomyopathy by improving mitochondrial function and decreasing premature apoptosis of cardiac cells, known as cardiomyocytes [87]. The unique anti-diabetic activities of resveratrol may partly be attributed to its ability to alter the composition of the gut microbiome, resulting in improved glucose homeostasis [85]. For example, resveratrol may help inhibit the growth of Bacteroidetes, a group of bacteria associated with processing fat, and influencing obesity conditions [85]. Furthermore, resveratrol may promote healthier gut microbiomes by inhibiting the production of small molecules by microbiota, such as trimethylamine-N-oxide (TMAO), which is linked to type-2 diabetes and obesity [85].

### 3.5. Anti-Virals

#### 3.5.1. HIV-1 and SARS

Black tea possesses anti-viral properties, generally attributed to its PPs, that specifically target the multiplication systems of viruses [78]. For example, theaflavins, a type of PP in black tea, inhibit the attachment of HIV-1 viruses to human cell targets, thereby demonstrating anti-HIV-1 properties [78,79,96]. HIV-1 attachment requires fusion of the viral glycoprotein and envelope to the target cell’s membrane and the envelope formation of a six-helical bundle to gain entry to the host cell. However, theaflavins may block the formation of HIV-1′s six-helix bundle, inhibiting viral entry into the host cell [79]. Additionally, theaflavin, along with other black tea PPs, also demonstrate effectiveness against the severe acute respiratory syndrome (SARS) virus where it inhibits viral multiplication mechanisms involving protease [79]. Certain types of tannins have been known to block the entry of viruses that utilize glycosaminoglycans (GAGs) on the surface of their target cell [117]. Two types of hydrolysable tannins with similar structures, chebulagic acid and punicalagin, can interact with viral glycoproteins and compete with GAGs [117]. The introduction of these tannins into the body may result in the covering of viral glycoproteins by tannins, thereby inhibiting viral attachment to GAGs on their target host cell and limiting subsequent viral replication and infection risks [117]. Viruses that target GAGs for cell entry include the human cytomegalovirus (HCMV), hepatitis C virus (HCV), dengue virus (DENV), measles virus (MV), and respiratory syncytial virus (RSV) [96,117]. However, the spread of certain GAG-targeting viruses, such as DENV and RSV, is not significantly affected by chebulagic acid and punicalagin treatment [117].

#### 3.5.2. Polyphenols and COVID-19

The impacts of COVID-19, caused by Severe Acute Respiratory Syndrome Coronavirus-2 (SARS-CoV-2), have proven to be detrimental worldwide. In conjunction with social distancing and mask-wearing, continued vaccination efforts are needed to limit the negative consequences of COVID-19. However, manufacturing and distributing vaccines for 7.8 billion people has been progressing very slowly amidst numerous challenges. Moreover, the emergence of new virus variants and the uncertainty of the long-term effectiveness of vaccines are also major concerns. Taking these concerns into consideration, investing in alternative strategies to manage the current pandemic, as well as future pandemics, should be a priority to researchers, health organizations, and policy makers. Here, we discuss the potential applications of PPs as preventative and symptomatic treatments for COVID-19. One advantage of PP treatment is that it can be administered quickly because it has already been approved for human consumption, making it a convenient method for treating COVID-19 symptoms and eliciting anti-viral effects [118].

SARS-CoV-2 is a single-stranded RNA virus that is structurally similar to SARS-CoV, a respiratory virus responsible for the 2003 coronavirus epidemic [119,120,121]. The minor symptoms of COVID-19 include fever, shortness of breath, coughing, and sore throat. However, more severe conditions may also arise such as pneumonia, severe acute respiratory syndrome, and other serious health problems [118,120]. PPs have been investigated for their potential to improve symptoms similar to that of COVID-19. Unlike many other medications used for treating these symptoms, the PPs have little to no adverse side effects in humans as demonstrated by Afsar and colleagues, who examined PPs as anti-inflammatory agents [122]. Furthermore, PPs were found to reduce inflammation more effectively than aspirin, but unlike aspirin, had no known side effects [122]. One of the most notable symptoms of COVID-19 is the prolonged cough, which is a naturally beneficial process that attempts to eject irritants from the respiratory tract. However, prolonged coughing is harmful and can lead to respiratory illness [123]. PPs, such as catechin, quercitrin, tannin, and ursolic acid, are known to alleviate coughing and sore throat symptoms associated with COVID-19 [123]. Many COVID-19 symptoms, including pneumonia and fever, may be attributed to viral binding of Toll-like receptors that release pro-inflammatory cytokines, leading to lung inflammation [124]. Various PPs are known to reduce inflammation by acting as free radical scavengers and suppressing mitogenic-activated protein kinase (MAPK) activity and nuclear-kappa signaling pathways, both of which lead to pro-inflammatory cytokine formation and iNOS mRNA expression [96,124,125,126,127]. Examples of PPs with anti-inflammatory and anti-cytokine properties include curcumin, resveratrol, flavonoids, and phenolic acids [124,125,126]. Furthermore, hyperthermia or fever, is caused by inflammatory processes and prostaglandin production, which leads to pyrexia by binding receptors in the brain, ultimately reducing heat loss and increasing heat production in the body [125,126,128]. In addition to their anti-inflammatory properties, PPs act as anti-pyretics by inhibiting prostaglandin synthesis and blocking cyclooxygenase enzyme activity and inflammatory mediators [125,126].

In addition to relieving COVID-19 symptoms, PPs may also be used as anti-viral agents to prevent viral infection. PPs demonstrate various strategies for inhibiting the replication of SARS-CoV-2 inside the human body [118,119], such as the inhibition of RNA dependent RNA polymerase (RdRp) [119,120], which is needed to replicate viral RNA for infection [120]. RdRp is an attractive anti-viral target candidate because humans do not possess any enzyme homologous to it, which allows protein-specific treatment to selectively target viral replication machinery [120]. This outcome may be attributed to the unique structural properties of PPs enabling them to interact with the active site of RdRp and bind with specific proteins and biomolecules [120]. For example, PPs found in black tea, such as theaflavin-3′-O-gallate (TF2a), theaflavin-3′-gallate (TF2b), theaflavin 3,3’-digallate (TF3), and epigallocatechin gallate (EGCG), have been known to inhibit RdRp [120]. Research also shows that each PP impacts RdRp differently [120], suggesting that diverse PP intake may positively affect human health. For example, TF2a impacts RdRp by reducing its flexibility, whereas TF3 reduces the expansion of proteins associated with the polymerase [120]. Another way in which PPs may act as anti-virals against COVID-19 is through the inhibition of Mpro [119,129]. Mpro, also known as 3 carbon-like proteases (3CLpro), is a cysteine protease in viruses that releases functional proteins from two overlapping open reading frames (ORF) [129]. Like RdRp, humans do not have an enzyme equivalent to Mpro, making this enzyme a potential anti-viral target [129]. The active site of Mpro consists of two regions (His41 and Cys145) that control the catalytic activity of the enzyme [129]. N3, a synthetic molecular reaction-based inhibitor, has shown potential to inhibit Mpro via binding to both His41 and Cys145 regions [130]. However, N3 may result in adverse side effects, giving rise to safety concerns that make it less ideal for human treatments [129]. On the other hand, many PPs found in green tea, known as catechins, also show inhibitory potential by binding to the His41 and Cys145 regions of Mpro [129]. These PPs, including epigallocatechin gallate (EGCG), epicatechin gallate (ECG), and gallocatechin-3-gallate (GCG), have similar bonding strength to that of N3 and are not considered harmful for human consumption [129].

Further research needs to be conducted in order to determine the amount of PP intake needed to be effective to treat symptoms or act as an anti-viral agent against SARS-CoV-2. However, it has been shown that a diverse set of PPs acting synergistically may reduce viral infectivity and offer potential for COVID-19 relief.

### 3.6. Bioavailability

Bioavailability refers to the amount of a substance that enters the body’s circulation and elicits an active effect [70]. PPs naturally have low bioavailability regardless of increasing dosage received orally [3,70]. This low bioavailability continually decreases as PPs proceed through the digestive system [3,131,132]. Although PPs can still provide health benefits when broken down by gut microbiota, like in the case of colorectal cancer, some PP structures are over-processed or go unused. During the digestive process, PPs may be metabolized via enzymatic reactions and can be bio-transformed into smaller, chemical intermediates by the gut microbiota [115]. It is proposed that only 5–10% of PPs intake enters blood circulation unchanged via the small intestine and becomes available for further biological functions [115,132], while the remaining PPs accumulate in the large intestine to be released into the lumen and may undergo further degradation and structural modifications [3,132]. However, despite their low bioavailability, PPs that enter blood circulation may elicit various beneficial effects, particularly if various PPs are ingested concurrently.

The bioavailability of PPs, as well as their active role in biological systems, largely depends upon the gut microbiome. For example, PPs have known impacts on lipid metabolism with the assistance of the gut microbiome [133]. Furthermore, findings suggest that gut microbiota transform certain PPs into bioactive forms that regulate lipid metabolism [133]. However, in addition to the microbiome shaping and altering the bioavailability of PPs in biological systems, PPs also benefit the gut microbiome itself [133,134,135]. For example, PPs intake can improve gut microbiome health, diversity, and resistance to pathogenic invaders [133,135]. As such, PPs intake has significant implications for human health purposes by enhancing gut microbiome health without serious side effects, unlike that of antibiotics and other drugs that have proven to be detrimental to gut microbiota [136].

While ingesting various PPs concurrently elicits beneficial health effects, overly high doses of PPs can lead to negative effects on human health. Much like the allelopathic effects in plant defense, increased consumption of phytochemicals may lead to adverse side effects in humans. For example, high PP intake has been shown to increase inflammation, damage DNA, and lead to pro-oxidative effects [131]. Furthermore, PPs possessing anti-cancer properties taken in high doses may induce tumor formation and the progression of cancerous cells [70]. Generally, most compounds may elicit either toxic or beneficial effects depending on the dose concentration [131]. The paradoxical nature of the dose-dependent responses to PPs illustrates the concept of hormesis which describes opposite effects observed for low and high-dose concentrations of a substance [137]. In the case of PPs, a low dose may elicit beneficial effects on the body, while higher doses may elicit adverse effects. 

Given this functional dilemma, investigating dose-dependent responses associated with phytochemical bioavailability and specific medicinal effects will be necessary in understanding their potential for dose-specific therapeutics. Furthermore, in conjunction with dose-dependent effects on bioavailability, investigating biotransformation processes via gut microbiota will further elucidate the fate of phytochemicals and their absorption potential for biological processes, which is crucial for their implementation in human therapeutic applications beyond the benefits derived from plant-based diets.

## 4. Challenges and Perspectives

As previously discussed, plants may alter PP concentrations and accumulate in specific plant tissues in response to environmental stressors. Utilizing this knowledge to help dictate agricultural-based strategies and practices may support higher crop yields and overall health via enhanced PPs levels in plants [52]. Implementing advanced biotechnologies, with an emphasis on phenotyping and genotyping, may help identify genes related to specific phytocompound production. Furthermore, the application of cutting-edge genome-editing techniques, such as CRISPR-Cas9, to develop crops with the capability to produce higher concentrations of PPs with plant tissue specificity and PPs type specificity. In cultivated crops, genetic diversity is typically low due to domestication, while wild species harbor higher genetic diversity. Therefore, there is a growing interest in investigating wild plant species due to their adaptation to harsh environmental conditions.

One of the main challenges regarding PP applications in human health is determining the proper dosage required to elicit an effective response while minimizing risks [138]. In a raw, plant-based diet, the bioactivity of PPs is dependent on numerous factors [138]. Furthermore, PPs may be converted into different metabolites that may elicit various effects [115]. Additionally, researchers have investigated how PPs can be modified to increase their bioactivity and duration in the body to provide long-term benefits [70]. Currently, the low oral bioavailability of PPs presents the most prevalent challenge in PP research and clinical applications. Despite their low bioavailability, PPs can still provide various human health benefits [115]. However, much research is still needed to understand the correlation between bioavailability and bioactivity to progress clinical studies. Furthermore, the mode of delivery and utilization by PPs needs to be investigated further to discern the potential trade-offs for different methods in terms of acquiring the most health benefits. For example, the intake of specific probiotics, in conjunction with PP intake, may decrease PPs absorption [132]. To date, PPs have mainly been investigated through in vitro studies. Emerging clinical trials will shed light on the potential applications of PPs in human health improvement and disease treatment.

## Figures and Tables

**Table 1:** Summary of the most common polyphenols synthesized in response to various environmental stressors.

Stressor Type	Stressor	Polyphenol(s) Induced	Reference
Abiotic	Drought	Flavonoids, Tannins, Stilbenes	[15,16]
	Temperature	Flavonoids, Tannins, Phenolic acids	[5,22,35,36]
	UV radiation	Flavonoid and Phenolic Acid	[5,40,43,44]
	Soil Salinity	Flavonoids and Tannins	[17,18]
	Heavy Metals	Phenolic Acids and Flavonoids	[31,48]
Biotic	Herbivores	Phenolic Acids, Tannins, Flavonoids	[12,19,20,21,59]
	Nematodes	Phenolic Acid, Lignin	[64,65,66,67]
	Competition	Phenolic Acid, Flavonoids, Tannins	[68]

**Table 2:** Plant polyphenols and human health. This table summarizes the role of various polyphenols in human health and the most common foods rich in specific polyphenols.

Polyphenol	Subcategory	Function	Source	Reference
Curcumin		Anti-inflammatory and antioxidant	Turmeric, curry powder	[76]
Flavonoids	Anthocyanins, Catechins, Theaflavins	Anti-inflammatory, anti-diabetic, anti-viral and antioxidant	Berries, grapes, grape seeds, strawberries, spinach	[70,77,78,79]
Lignans		Anti-inflammatory, antioxidant and anti-tumor	Sesame and flax seeds, grains, nuts, and soy	[80,81,82]
Phenolic Acids		Anti-inflammatory, antioxidant and anti-tumor	Wheat, barley, oat and rye	[83,84]
Stilbenes	Resveratrol	Anti-inflammatory, anti-diabetic, antioxidant	Wine, berries, peanuts, tea	[85,86,87]
Tannins	Chebulagic Acid and Punicalagin	Anti-inflammatory, anti-diabetic, antioxidant	Coffee, tea, wine, grapes, apricots, barley, peaches, dry fruits, mint, basil, rosemary, pomegranate, strawberries clove, rice, oat, rye	[87,88,89]

## Data Availability

Not applicable.

## References

[1] Short E.E., Caminade C., Thomas B.N. (2017). Climate change contribution to the emergence or re-emergence of parasitic diseases. Infect. Dis. Res. Treat..

[2] Huang Y., Xiao D., Burton-Freeman B.M., Edirisinghe I. (2016). Chemical changes of bioactive phytochemicals during thermal processing. Reference Module in Food Science.

[3] Pandey K.B., Rizvi S.I. (2009). Plant polyphenols as dietary antioxidants in human health and disease. Oxidative Med. Cell. Longev..

[4] Wang J., Xu J., Gong X., Yang M., Zhang C., Li M. (2019). Biosynthesis, chemistry, and pharmacology of polyphenols from chinese salvia species: A review. Molecules.

[5] Sharma A., Shahzad B., Rehman A., Bhardwaj R., Landi M., Zheng B. (2019). Response of phenylpropanoid pathway and the role of polyphenols in plants under abiotic stress. Molecules.

[6] Kallscheuer N., Vogt M., Marienhagen J. (2016). A novel synthetic pathway enables microbial production of polyphenols independent from the endogenous aromatic amino acid metabolism. ACS Synth. Biol..

[7] Caretto S., Linsalata V., Colella G., Mita G., Lattanzio V. (2015). Carbon fluxes between primary metabolism and phenolic pathway in plant tissues under stress. Int. J. Mol. Sci..

[8] Pereira A. (2016). plant abiotic stress challenges from the changing environment. Front. Plant Sci..

[9] Jiang S., Weng B., Liu T., Su Y., Liu J., Lu H., Yan C. (2017). Response of phenolic metabolism to cadmium and phenanthrene and its influence on pollutant translocations in the mangrove plant *Aegiceras corniculatum* (L.) *Blanco* (Ac). Ecotoxicol. Environ. Saf..

[10] Milke L., Aschenbrenner J., Marienhagen J., Kallscheuer N. (2018). Production of plant-derived polyphenols in microorganisms: Current state and perspectives. Appl. Microbiol. Biotechnol..

[11] Cui N., Lu H., Wang T., Zhang W., Kang L., Cui F. (2019). Armet, an aphid effector protein, induces pathogen resistance in plants by promoting the accumulation of salicylic acid. Philos. Trans. R. Soc. B Biol. Sci..

[12] Czerniewicz P., Sytykiewicz H., Durak R., Borowiak-Sobkowiak B., Chrzanowski G. (2017). Role of phenolic compounds during antioxidative responses of winter triticale to aphid and beetle attack. Plant Physiol. Biochem..

[13] Body M.J.A., Neer W.C., Vore C., Lin C.-H., Vu D.C., Schultz J.C., Cocroft R.B., Appel H.M. (2019). Caterpillar chewing vibrations cause changes in plant hormones and volatile emissions in arabidopsis thaliana. Front. Plant Sci..

[14] Li Y.-P., Feng Y.-L., Chen Y.-J., Tian Y.-H. (2015). Soil microbes alleviate allelopathy of invasive plants. Sci. Bull..

[15] Pinasseau L., Vallverdú-Queralt A., Verbaere A., Roques M., Meudec E., Le Cunff L., Péros J.-P., Ageorges A., Sommerer N., Boulet J.-C. (2017). Cultivar diversity of grape skin polyphenol composition and changes in response to drought investigated by LC-MS based metabolomics. Front. Plant Sci..

[16] Caser M., D’Angiolillo F., Chitarra W., Lovisolo C., Ruffoni B., Pistelli L., Pistelli L., Scariot V. (2018). Ecophysiological and phytochemical responses of *Salvia sinaloensis* Fern. to drought stress. Plant Growth Regul..

[17] Slama I., M’Rabet R., Ksouri R., Talbi O., Debez A., Abdelly C. (2017). Effects of salt treatment on growth, lipid membrane peroxidation, polyphenol content, and antioxidant activities in leaves of *Sesuvium portulacastrum* L.. Arid Land Res. Manag..

[18] Abideen Z., Qasim M., Rasheed A., Adnan M.Y., Gul B., Khan M.A. (2015). Antioxidant activity and polyphenol content of Phragmites karka under saline conditions. Pak. J. Bot..

[19] Rani P.U., Sambangi P., Sandhyarani K. (2017). Impact of plant phenolics as semiochemicals on the performance of trichogramma chilonis Ishii. J. Insect Behav..

[20] Kariyat R.R., Gaffoor I., Sattar S., Dixon C.W., Frock N., Moen J., De Moraes C.M., Mescher M.C., Thompson G.A., Chopra S. (2019). Sorghum 3-deoxyanthocyanidin flavonoids confer resistance against corn leaf aphid. J. Chem. Ecol..

[21] Chu B., Zhang S., Wang L., Zhu X.-Z., Luo J.-Y., Wang C.-Y., Lü L.-M., Cui J.-J. (2017). Genetic regulation of defence responses in cotton to insect herbivores. AoB PlANS.

[22] Martinez V., Mestre T.C., Rubio F., Girones-Vilaplana A., Moreno-Fernández D.A., Mittler R., Rivero R.M. (2016). Accumulation of flavonols over hydroxycinnamic acids favors oxidative damage protection under abiotic stress. Front. Plant Sci..

[23] Aninbon C., Jogloy S., Vorasoot N., Patanothai A., Nuchadomrong S., Senawong T. (2016). Effect of end of season water deficit on phenolic compounds in peanut genotypes with different levels of resistance to drought. Food Chem..

[24] Kaur L., Zhawar V.K. (2015). Phenolic parameters under exogenous ABA, water stress, salt stress in two wheat cultivars varying in drought tolerance. Indian J. Plant Physiol..

[25] Griesser M., Weingart G., Schoedl-Hummel K., Neumann N., Becker M., Varmuza K., Liebner F., Schuhmacher R., Forneck A. (2015). Severe drought stress is affecting selected primary metabolites, polyphenols, and volatile metabolites in grapevine leaves (*Vitis vinifera* cv. Pinot noir). Plant Physiol. Biochem..

[26] Ortiz N., Armada E., Duque E., Roldan A., Azcón R. (2015). Contribution of arbuscular mycorrhizal fungi and/or bacteria to enhancing plant drought tolerance under natural soil conditions: Effectiveness of autochthonous or allochthonous strains. J. Plant Physiol..

[27] Tombesi S., Nardini A., Frioni T., Soccolini M., Zadra C., Farinelli D., Poni S., Palliotti A. (2015). Stomatal closure is induced by hydraulic signals and maintained by ABA in drought-stressed grapevine. Sci. Rep..

[28] Hazzoumi Z., Moustakime Y., Elharchli E.H., Joutei K.A. (2015). Effect of arbuscular mycorrhizal fungi (AMF) and water stress on growth, phenolic compounds, glandular hairs, and yield of essential oil in basil (*Ocimum gratissimum* L). Chem. Biol. Technol. Agric..

[29] Akhtar W., Mahmood T. (2017). Response of rice polyphenol oxidase promoter to drought and salt stress. Pak. J. Bot..

[30] Varela M.C., Arslan I., Reginato M.A., Cenzano A.M., Luna M.V. (2016). Phenolic compounds as indicators of drought resistance in shrubs from Patagonian shrublands (Argentina). Plant Physiol. Biochem..

[31] Maslennikov P.V., Chupakhina G.N., Skrypnik L.N., Feduraev P.V., Melnik A.S. (2018). Assessment of the antioxidant potential of plants in urban ecosystems under conditions of anthropogenic pollution of soils. Russ. J. Ecol..

[32] Szymańska R., Ślesak I., Orzechowska A., Kruk J. (2017). Physiological and biochemical responses to high light and temperature stress in plants. Environ. Exp. Bot..

[33] Awasthi R., Bhandari K., Nayyar H. (2015). Temperature stress and redox homeostasis in agricultural crops. Front. Environ. Sci..

[34] Świeca M. (2015). Elicitation with abiotic stresses improves pro-health constituents, antioxidant potential and nutritional quality of lentil sprouts. Saudi J. Biol. Sci..

[35] Qiu Z., Wang X., Gao J., Guo Y., Huang Z., Du Y. (2016). The tomato hoffman’s anthocyaninless gene encodes a bhlh transcription factor involved in anthocyanin biosynthesis that is developmentally regulated and induced by low temperatures. PLoS ONE.

[36] Wang L., Shan T., Xie B., Ling C., Shao S., Jin P., Zheng Y. (2019). Glycine betaine reduces chilling injury in peach fruit by enhancing phenolic and sugar metabolisms. Food Chem..

[37] Klem K., Holub P., Štroch M., Nezval J., Špunda V., Tříska J., Jansen M., Robson T.M., Urban O. (2015). Ultraviolet and photosynthetically active radiation can both induce photoprotective capacity allowing barley to overcome high radiation stress. Plant Physiol. Biochem..

[38] Barnes P.W., Tobler M.A., Keefover-Ring K., Flint S.D., Barkley A.E., Ryel R.J., Lindroth R.L. (2016). Rapid modulation of ultraviolet shielding in plants is influenced by solar ultraviolet radiation and linked to alterations in flavonoids. Plant Cell Environ..

[39] Gutierrez E., García-Villaraco A., Lucas J.A., Gradillas A., Gutierrez-Mañero F.J., Ramos-Solano B. (2017). Transcriptomics, targeted metabolomics and gene expression of blackberry leaves and fruits indicate flavonoid metabolic flux from leaf to red fruit. Front. Plant Sci..

[40] Wu G., Bornman J.F., Bennett S.J., Clarke M., Fang Z., Johnson S. (2017). Individual polyphenolic profiles and antioxidant activity in sorghum grains are influenced by very low and high solar UV radiation and genotype. J. Cereal Sci..

[41] Barnes P.W., Flint S.D., Ryel R.J., Tobler M.A., Barkley A.E., Wargent J.J. (2015). Rediscovering leaf optical properties: New insights into plant acclimation to solar UV radiation. Plant Physiol. Biochem..

[42] Fu B., Ji X., Zhao M., He F., Wang X., Wang Y., Liu P., Niu L. (2016). The influence of light quality on the accumulation of flavonoids in tobacco ( Nicotiana tabacum L.) leaves. J. Photochem. Photobiol. B Biol..

[43] Henry-Kirk R.A., Plunkett B., Hall M., McGhie T., Allan A.C., Wargent J.J., Espley R. (2018). Solar UV light regulates flavonoid metabolism in apple (Malus x domestica). Plant Cell Environ..

[44] Xu Y., Charles M.T., Luo Z., Mimee B., Veronneau P.-Y., Rolland D., Roussel D. (2017). Preharvest ultraviolet C irradiation increased the level of polyphenol accumulation and flavonoid pathway gene expression in strawberry fruit. J. Agric. Food Chem..

[45] Zrig A., Tounekti T., AbdElgawad H., Hegab M.M., Ali S.O., Khemira H. (2016). Essential oils, amino acids and polyphenols changes in salt-stressed Thymus vulgaris exposed to open–field and shade enclosure. Ind. Crop. Prod..

[46] Qi J., Zhang M., Lu C., Hettenhausen C., Tan Q., Cao G., Zhu X., Wu G., Wu J. (2018). Ultraviolet-B enhances the resistance of multiple plant species to lepidopteran insect herbivory through the jasmonic acid pathway. Sci. Rep..

[47] Stagnari F., Galieni A., D’Egidio S., Falcinelli B., Pagnani G., Pace R., Pisante M., Benincasa P. (2017). Effects of sprouting and salt stress on polyphenol composition and antiradical activity of einkorn, emmer and durum wheat. Ital. J. Agron..

[48] Gratão P., Alves L., Lima L. (2019). Heavy Metal Toxicity and Plant Productivity: Role of Metal Scavengers. Plant-Metal Interactions.

[49] Wang C., Li W., Yang Z., Chen Y., Shao W., Ji J. (2015). An invisible soil acidification: Critical role of soil carbonate and its impact on heavy metal bioavailability. Sci. Rep..

[50] Chen D., Liu X., Bian R., Cheng K., Zhang X., Zheng J., Joseph S., Crowley D., Pan G., Li L. (2018). Effects of biochar on availability and plant uptake of heavy metals – A meta-analysis. J. Environ. Manag..

[51] Xu H.-Q., Zhang J.-E., Ouyang Y., Lin L., Quan G.-M., Zhao B.-L., Yu J.-Y. (2015). Effects of simulated acid rain on microbial characteristics in a lateritic red soil. Environ. Sci. Pollut. Res..

[52] Heimler D., Romani A., Ieri F. (2017). Plant polyphenol content, soil fertilization and agricultural management: A review. Eur. Food Res. Technol..

[53] Gautam M., Elhiti M., Fomsgaard I.S. (2018). Maize root culture as a model system for studying azoxystrobin biotransformation in plants. Chemosphere.

[54] Pan Q., Shikano I., Hoover K., Liu T.-X., Felton G.W. (2019). Pathogen-mediated tritrophic interactions: Baculovirus-challenged caterpillars induce higher plant defenses than healthy caterpillars. J. Chem. Ecol..

[55] Austel N., Eilers E.J., Meiners T., Hilker M. (2015). Elm leaves ‘warned’ by insect egg deposition reduce survival of hatching larvae by a shift in their quantitative leaf metabolite pattern. Plant Cell Environ..

[56] Ibanez F., Bang W.Y., Lombardini L., Cisneros-Zevallos L. (2019). Solving the controversy of healthier organic fruit: Leaf wounding triggers distant gene expression response of polyphenol biosynthesis in strawberry fruit (*Fragaria* x *ananassa*). Sci. Rep..

[57] Tayal M., Somavat P., Rodriguez I., Martinez L., Kariyat R. (2020). Cascading effects of polyphenol-rich purple corn pericarp extract on pupal, adult, and offspring of tobacco hornworm (*Manduca sexta* L.). Commun. Integr. Biol..

[58] Joshi N., Nautiyal P., Papnai G., Supyal V., Singh K. (2019). Render a sound dose: Effects of implementing acoustic frequencies on plants’ physiology, biochemistry and genetic makeup. IJCS.

[59] Akšić M.M.F., Dabić D.Č., Gašić U., Zec G.N., Vulić T.B., Tesic Z., Natić M. (2015). Polyphenolic profile of Pear leaves with different resistance to Pear Psylla (*Cacopsylla pyri*). J. Agric. Food Chem..

[60] Liu X., Vrieling K., Klinkhamer P.G. (2017). Interactions between plant metabolites affect herbivores: A study with pyrrolizidine alkaloids and chlorogenic acid. Front. Plant Sci..

[61] Kumar P., Vargas-Ortiz E., Garrido E., Poveda K., Jander G. (2016). Potato tuber herbivory increases resistance to aboveground lepidopteran herbivores. Oecologia.

[62] Pentzold S., Burse A., Boland W. (2017). Contact chemosensation of phytochemicals by insect herbivores. Nat. Prod. Rep..

[63] Lu K., Cheng Y., Li Y., Li W., Zeng R., Song Y. (2021). Phytochemical flavone confers broad-spectrum tolerance to insecticides in spodoptera litura by activating ROS/CncC-mediated xenobiotic detoxification pathways. J. Agric. Food Chem..

[64] Bali S., Kaur P., Sharma A., Ohri P., Bhardwaj R., Wijaya L., Ahmad P., Alyemeni M.N. (2017). Jasmonic acid-induced tolerance to root-knot nematodes in tomato plants through altered photosynthetic and antioxidative defense mechanisms. Protoplasma.

[65] Gao X., Zhang S., Zhao X., Wu Q. (2018). Potassium-induced plant resistance against soybean cyst nematode via root exudation of phenolic acids and plant pathogen-related genes. PLoS ONE.

[66] Patel V.S., Shukla Y., Dhruve J. (2017). Influence of root knot nematode (*Meloidogyne spp*.) on phenolic acid profile in root of tomato (*Solanum lycopersicum* L.). Int. J. Curr. Microbiol. Appl. Sci..

[67] Xiao Z., Liu M., Jiang L., Chen X., Griffiths B., Li H., Hu F. (2016). Vermicompost increases defense against root-knot nematode (*Meloidogyne incognita*) in tomato plants. Appl. Soil Ecol..

[68] Sant’Anna V., Biondo E., Kolchinski E.M., da Silva L.F.S., Corrêa A.P.F., Bach E., Brandelli A. (2017). Total polyphenols, antioxidant, antimicrobial and allelopathic activities of spend coffee ground aqueous extract. Waste Biomass Valorization.

[69] Baličević R., Ravlić M., Živković T. (2015). Allelopathic effect of invasive species giant goldenrod (solidago gigantea ait.) on crops and weedS. Herbol. Int. J. Weed Res. Control..

[70] Brglez Mojzer E., Knez Hrnčič M., Škerget M., Knez Ž., Bren U. (2016). Polyphenols: Extraction methods, antioxidative action, bioavailability and anticarcinogenic effects. Molecules.

[71] Abd-Elgawad A.M., ElShamy A.I., Al-Rowaily S.L., El-Amier Y.A. (2019). Habitat affects the chemical profile, allelopathy, and antioxidant properties of essential oils and phenolic enriched extracts of the invasive plant *Heliotropium Curassavicum*. Plants.

[72] Islam A.K.M.M., Widhalm J.R. (2020). Agricultural uses of juglone: Opportunities and challenges. Agronomy.

[73] Macías F.A., Mejías F.J.R., Molinillo J.M.G. (2019). Recent advances in allelopathy for weed control: From knowledge to applications. Pest Manag. Sci..

[74] Mahmood K., Khan M.B., Song Y.Y., Ijaz M., Luo S.M., Zeng R. (2013). UV-irradiation enhances rice allelopathic potential in rhizosphere soil. Plant Growth Regul..

[75] Vos I.A., Emoritz L., Pieterse C., Van Wees S.C.M. (2015). Impact of hormonal crosstalk on plant resistance and fitness under multi-attacker conditions. Front. Plant Sci..

[76] Hewlings S.J., Kalman D.S. (2017). Curcumin: A review of its effects on human health. Foods.

[77] Shukitt-Hale B., Lau F.C., Joseph J.A. (2008). Berry fruit supplementation and the aging brain. J. Agric. Food Chem..

[78] Yang Z.-F., Bai L.-P., Huang W.-B., Li X.-Z., Zhao S.-S., Zhong N.-S., Jiang Z.-H. (2014). Comparison of in vitro antiviral activity of tea polyphenols against influenza A and B viruses and structure–activity relationship analysis. Fitoterapia.

[79] Lin L.-T., Chen T.-Y., Lin S.-C., Chung C.-Y., Lin T.-C., Wang G.-H., Anderson R., Lin C.-C., Richardson C.D. (2013). Broad-spectrum antiviral activity of chebulagic acid and punicalagin against viruses that use glycosaminoglycans for entry. BMC Microbiol..

[80] Huang X.-X., Bai M., Zhou L., Lou L.-L., Liu Q.-B., Zhang Y., Li L.-Z., Song S.-J. (2015). Food byproducts as a new and cheap source of bioactive compounds: Lignans with antioxidant and anti-inflammatory properties from *Crataegus Pinnatifida* seeds. J. Agric. Food Chem..

[81] McCann S.E., Thompson L.U., Nie J., Dorn J., Trevisan M., Shields P.G., Ambrosone C.B., Edge S.B., Li H.-F., Kasprzak C. (2009). Dietary lignan intakes in relation to survival among women with breast cancer: The Western New York Exposures and Breast Cancer (WEB) study. Breast Cancer Res. Treat..

[82] Rodríguez-García C., Sánchez-Quesada C., Toledo E., Delgado-Rodríguez M., Gaforio J.J. (2019). Naturally lignan-rich foods: A dietary tool for health promotion?. Molecules.

[83] Călinoiu L.F., Vodnar D.C. (2018). Whole grains and phenolic acids: A review on bioactivity, functionality, health benefits and bioavailability. Nutrients.

[84] Koushik A., Hunter D.J., Spiegelman D., Beeson W.L., Brandt P.A.V.D., Buring J.E., Calle E.E., Cho E., Fraser G.E., Freudenheim J.L. (2007). Fruits, vegetables, and colon cancer risk in a pooled analysis of 14 cohort studies. J. Natl. Cancer Inst..

[85] Springer M., Moco S. (2019). Resveratrol and its human metabolites-effects on metabolic health and obesity. Nutrients.

[86] Breuss J.M., Atanasov A.G., Uhrin P. (2019). Resveratrol and its effects on the vascular system. Int. J. Mol. Sci..

[87] Galiniak S., Aebish D., Bartusik-Aebisher D. (2019). Health benefits of resveratrol administration. In Acta Biochim. Pol..

[88] Laddha A.P., Kulkarni Y.A. (2019). Tannins and vascular complications of Diabetes: An update. Phytomedicine.

[89] Varatharajan R., Sattar M.Z.A., Chung I., Abdulla M.A., Kassim N.M., Abdullah N.A. (2013). Antioxidant and pro-oxidant effects of oil palm (*Elaeis guineensis*) leaves extract in experimental diabetic nephropathy: A duration-dependent outcome. BMC Complement. cltern. Med..

[90] Rajendran P., Nandakumar N., Rengarajan T., Palaniswami R., Gnanadhas E.N., Lakshminarasaiah U., Gopas J., Nishigaki I. (2014). Antioxidants and human diseases. Clin. Chim. Acta.

[91] Merlin J.P.J., Rupasinghe H.P.V., Dellaire G., Murphy K. (2021). Role of dietary antioxidants in p53-mediated cancer chemoprevention and tumor suppression. Oxidative Med. Cell. Longev..

[92] Xu D.-P., Li Y., Meng X., Zhou T., Zhou Y., Zheng J., Zhang J.-J., Li H.-B. (2017). Natural antioxidants in foods and medicinal plants: Extraction, assessment and resources. Int. J. Mol. Sci..

[93] Guzzetti L., Panzeri D., Ulaszewska M., Sacco G., Forcella M., Fusi P., Tommasi N., Fiorini A., Campone L., Labra M. (2021). Assessment of dietary bioactive phenolic compounds and agricultural sustainability of an African leafy vegetable *Corchorus Olitorius* L.. Front. Nutr..

[94] Bosso H., Barbalho S.M., Goulart R.D.A., Otoboni A.M.M.B. (2021). Green coffee: Economic relevance and a systematic review of the effects on human health. Crit. Rev. Food Sci. Nutr..

[95] Xia N., Daiber A., Förstermann U., Li H. (2016). Antioxidant effects of resveratrol in the cardiovascular system. Br. J. Pharmacol..

[96] Jibril F.I., Hilmi A.B.M., Manivannan L. (2019). Isolation and characterization of polyphenols in natural honey for the treatment of human diseases. Bull. Natl. Res. Cent..

[97] Grzesik M., Naparło K., Bartosz G., Sadowska-Bartosz I. (2018). Antioxidant properties of catechins: Comparison with other antioxidants. Food Chem..

[98] Siracusa R., Monaco F., D’Amico R., Genovese T., Cordaro M., Interdonato L., Gugliandolo E., Peritore A., Crupi R., Cuzzocrea S. (2021). Epigallocatechin-3-gallate modulates postoperative pain by regulating biochemical and molecular pathways. Int. J. Mol. Sci..

[99] Stanislaus N.N., Ibrahim S.M., Usman P.U., Sa’adiya H.H., Garba M.M., Sa’ad Toyin A., Taibat Moji B.O., Ndifor A.R., Osas E.G., Oyetunji S.A. (2021). Antimicrobial and antioxidant activity of catechin-3-o-rhamnoside isolated from the stem bark of *Lannea kerstingii* Engl. and K. Krause (*Anacardiaceae*). Pak. J. Pharm. Sci..

[100] Fidelis M., Santos J.S., Escher G.B., Rocha R.S., Cruz A.G., Cruz T.M., Marques M.B., Nunes J.B., Carmo M.A.V.D., de Almeida L.A. (2021). Polyphenols of jabuticaba [Myrciaria jaboticaba (Vell.) O.Berg] seeds incorporated in a yogurt model exert antioxidant activity and modulate gut microbiota of 1,2-dimethylhydrazine-induced colon cancer in rats. Food Chem..

[101] Tedesco I., Spagnuolo C., Russo G., Russo M., Cervellera C., Moccia S. (2021). The pro-oxidant activity of red wine polyphenols induces an adaptive antioxidant response in human erythrocytes. Antioxidants.

[102] Forman H.J., Davies K.J., Ursini F. (2014). How do nutritional antioxidants really work: Nucleophilic tone and para-hormesis versus free radical scavenging in vivo. Free Radic. Biol. Med..

[103] Chambers C.S., Biedermann D., Valentová K., Petrásková L., Viktorová J., Kuzma M., Křen V. (2019). Preparation of retinoyl-flavonolignan hybrids and their antioxidant properties. Antioxidants.

[104] Han R.-M., Zhang J.-P., Skibsted L.H. (2012). Reaction dynamics of flavonoids and carotenoids as antioxidants. Molecules.

[105] Okuyama S., Sawamoto A., Nakajima M., Furukawa Y. (2021). The search for citrus components with neuroprotective and anti-dementia effects in the mouse brain. Yakugaku Zasshi J. Pharm. Soc. Jpn..

[106] Ayaz M., Sadiq A., Junaid M., Ullah F., Ovais M., Ullah I., Ahmed J., Shahid M. (2019). Flavonoids as prospective neuroprotectants and their therapeutic propensity in aging associated neurological disorders. Front. Aging Neurosci..

[107] Domínguez-García S., Gómez-Oliva R., Geribaldi-Doldán N., Hierro-Bujalance C., Sendra M., Ruiz F.A., Carrascal L., Macías-Sánchez A.J., Verástegui C., Hernández-Galán R. (2021). Effects of classical PKC activation on hippocampal neurogenesis and cognitive performance: Mechanism of action. Neuropsychopharmacology.

[108] Sun J., Zhou X., Wu J., Xiao R., Chen Y., Lu Y., Lang H. (2021). Ligustilide enhances hippocampal NSCs activation to restore cognitive function in the context of postoperative cognitive dysfunction. Eur. J. Neurosci..

[109] Behl T., Mehta K., Sehgal A., Singh S., Sharma N., Ahmadi A., Arora S., Bungau S. (2021). Exploring the role of polyphenols in rheumatoid arthritis. Crit. Rev. Food Sci. Nutr..

[110] Jiang K., Yang J., Xue G., Dai A., Wu H. (2021). Fisetin ameliorates the inflammation and oxidative stress in lipopolysaccharide-induced endometritis. J. Inflamm. Res..

[111] Loria V., Dato I., Graziani F., Biasucci L.M. (2008). Myeloperoxidase: A new biomarker of inflammation in ischemic heart disease and acute coronary syndromes. Mediat. Inflamm..

[112] Calniquer G., Khanin M., Ovadia H., Linnewiel-Hermoni K., Stepensky D., Trachtenberg A., Sedlov T., Braverman O., Levy J., Sharoni Y. (2021). Combined effects of carotenoids and polyphenols in balancing the response of skin cells to uv irradiation. Molecules.

[113] Zálešák F., Bon D.J.-Y.D., Pospíšil J. (2019). Lignans and Neolignans: Plant secondary metabolites as a reservoir of biologically active substances. Pharmacol. Res..

[114] Rattanaburee T., Tipmanee V., Tedasen A., Thongpanchang T., Graidist P. (2020). Inhibition of CSF1R and AKT by (±)-kusunokinin hinders breast cancer cell proliferation. Biomed. Pharmacother..

[115] Luca S.V., Macovei I., Bujor A., Miron A., Skalicka-Woźniak K., Aprotosoaie A.C., Trifan A. (2020). Bioactivity of Dietary Polyphenols: The Role of Metabolites.

[116] Bahrami A., Jafari S., Rafiei P., Beigrezaei S., Sadeghi A., Hekmatdoost A., Rashidkhani B., Hejazi E. (2019). Dietary intake of polyphenols and risk of colorectal cancer and adenoma–A case-control study from Iran. Complement. Ther. Med..

[117] Sharma V., Rao L.J.M. (2009). A thought on the biological activities of black tea. Crit. Rev. Food Sci. Nutr..

[118] Chojnacka K., Witek-Krowiak A., Skrzypczak D., Mikula K., Młynarz P. (2020). Phytochemicals containing biologically active polyphenols as an effective agent against COVID-19-inducing coronavirus. J. Funct. Foods.

[119] Mhatre S., Srivastava T., Naik S., Patravale V. (2021). Antiviral activity of green tea and black tea polyphenols in prophylaxis and treatment of COVID-19: A review. Phytomedicine.

[120] Singh S., Sk F., Sonawane A., Kar P., Sadhukhan S. (2020). Plant-derived natural polyphenols as potential antiviral drugs against SARS-CoV-2 via RNA-dependent RNA polymerase (RdRp) inhibition: An in-silico analysis. J. Biomol. Struct. Dyn..

[121] Giannis D., Ziogas I.A., Gianni P. (2020). Coagulation disorders in coronavirus infected patients: COVID-19, SARS-CoV-1, MERS-CoV and lessons from the past. J. Clin. Virol..

[122] Afsar T., Khan M.R., Razak S., Ullah S., Mirza B. (2015). Antipyretic, anti-inflammatory and analgesic activity of Acacia hydaspica R. Parker and its phytochemical analysis. BMC Complement. Altern. Med..

[123] Sultana S., Khan A., Safhi M., Alhazmi H. (2016). Cough suppressant herbal drugs: A review. Int. J. Pharm. Sci. Invent..

[124] Muzio L.L., Bizzoca M.E., Ravagnan G. (2020). New intriguing possibility for prevention of coronavirus pneumonitis: Natural purified polyphenols. Oral Dis..

[125] Ghareeb M.A., Sobeh M., Rezq S., El-Shazly A.M., Mahmoud M.F., Wink M. (2018). HPLC-ESI-MS/MS profiling of polyphenolics of a leaf extract from alpinia zerumbet (*Zingiberaceae*) and its anti-inflammatory, anti-nociceptive, and antipyretic activities in vivo. Molecules.

[126] Shanmugam S., Murugaiyan I., Lima B.D.S., Serafini M.R., Araújo A.A.D.S., Narain N., Quintans L., Thangaraj P. (2019). HPLC–DAD–MS identification of polyphenols from Passiflora leschenaultii and determination of their antioxidant, analgesic, anti-inflammatory and antipyretic properties. Arab. J. Chem..

[127] Shen C.-Y., Jiang J.-G., Huang C.-L., Zhu W., Zheng C.-Y. (2017). polyphenols from blossoms of citrus aurantiuml. var.amaraengl. show significant anti-complement and anti-inflammatory effects. J. Agric. Food Chem..

[128] Milton A.S. (1998). Prostaglandins and Fever: Progress in Brain Ressarch.

[129] Ghosh R., Chakraborty A., Biswas A., Chowdhuri S. (2020). Evaluation of green tea polyphenols as novel corona virus (SARS-CoV-2) main protease (Mpro) inhibitors—anin silicodocking and molecular dynamics simulation study. J. Biomol. Struct. Dyn..

[130] Mittal L., Kumari A., Srivastava M., Singh M., Asthana S. (2021). Identification of potential molecules against COVID-19 main protease through structure-guided virtual screening approach. J. Biomol. Struct. Dyn..

[131] Granato D., Mocan A., Câmara J. (2020). Is a higher ingestion of phenolic compounds the best dietary strategy? A scientific opinion on the deleterious effects of polyphenols in vivo. Trends Food Sci. Technol..

[132] Kumar Singh A., Cabral C., Kumar R., Ganguly R., Kumar Rana H., Gupta A., Lauro M.R., Carbone C., Reis F., Pandey A.K. (2019). Beneficial effects of dietary polyphenols on gut microbiota and strategies to improve delivery efficiency. Nutrients.

[133] Ma J., Zheng Y., Tang W., Yan W., Nie H., Fang J., Liu G. (2020). Dietary polyphenols in lipid metabolism: A role of gut microbiome. Anim. Nutr..

[134] Du H., Wang Q., Li T., Ren D., Yang X. (2021). Grape seed proanthocyanidins reduced the overweight of C57BL/6J mice through modulating adipose thermogenesis and gut microbiota. Food Funct..

[135] Rodríguez-Daza M.C., Pulido-Mateos E.C., Lupien-Meilleur J., Guyonnet D., Desjardins Y., Roy D. (2021). Polyphenol-mediated gut microbiota modulation: Toward prebiotics and further. Front. Nutr..

[136] Vich Vila A., Collij V., Sanna S., Sinha T., Imhann F., Bourgonje A., Mujagic Z., Jonkers D.M.A.E., Masclee A.A.M., Fu J. (2020). Impact of commonly used drugs on the composition and metabolic function of the gut microbiota. Nat. Commun..

[137] Holbrook J.B., Mitcham C. (2015). Ethics, Science, Technology, and Engineerin: A Global Resource.

[138] Williamson G. (2017). The role of polyphenols in modern nutrition. Nutr. Bull..

